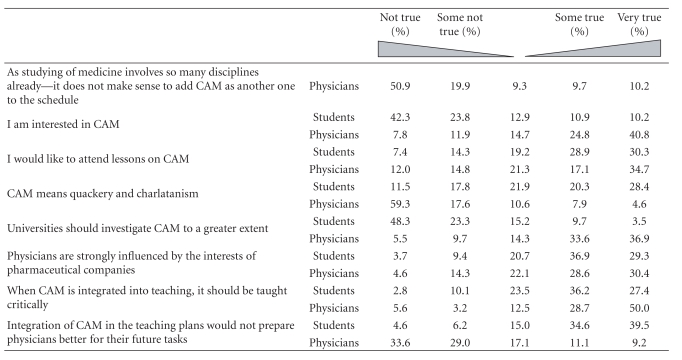# Complementary and Alternative Medicine: Comparison of Current Knowledge, Attitudes and Interest among German Medical Students and Doctors

**DOI:** 10.1093/ecam/nen079

**Published:** 2011-06-18

**Authors:** Karsten Münstedt, Hildegard Harren, Richard von Georgi, Andreas Hackethal

**Affiliations:** ^1^Department of Obstetrics and Gynecology, Justus-Liebig-University of Giessen, Klinikstrasse 32, D-35385 Giessen, Germany; ^2^Institute for Music Sciences, Justus-Liebig-University of Giessen, Klinikstrasse 32, D-35385 Giessen, Germany

## Abstract

Although it has been agreed that complementary and alternative medicine (CAM) should be included in the German medical curriculum, there is no consensus on which methods and how it should be taught. This study aimed to assess needs for CAM education by evaluating current knowledge, attitudes and interests of medical students, general physicians and gynecologists. Two instruments based on established and validated questionnaires were developed. One was given to seventh semester medical students and the other to office-based doctors. Data were analyzed by bivariate correlation and cross-tabulation. Altogether 550 questionnaires were distributed—280 to doctors and 270 to medical students. Completed questionnaires were returned by 80.4% of students and 78.2% of doctors. Although 73.8% (160/219) of doctors and 40% (87/217) of students had already informed themselves about CAM, neither group felt that they knew much about CAM. Doctors believed that CAM was most useful in general medicine, supportive oncology, pediatrics, dermatology and gynecology, while students believed that dermatology, general medicine, psychiatry and rheumatology offered opportunities; both recommended that CAM should be taught in these areas. Both groups believed that CAM should be included in medical education; however, they believed that CAM needed more investigation and should be taught “critically". German doctors and students would like to be better informed about CAM. An approach which teaches fundamental competences to students, chooses specific content based on evidence, demographics and medical conditions and provides students with the skills they need for future learning should be adopted.

## 1. Introduction

Alternative and complimentary medicine (CAM) is practiced all over the world. Well-known examples of medical specialties in which CAM therapies are frequently used are oncology and obstetrics [1, 2]. In the past, medical schools tended to focus on the question of whether CAM should be taught to medical students at all, but the debate has now moved on to how much, which methods and when CAM should be taught [3, 4].

Gaylord et al. [5] have described the reasons for developing CAM educational programs as part of medical education in the United States. These include.

- The prevalence of CAM and the increase in its use.
- The need to enhance the safety of CAM use and interactions with conventional medical care.
- CAM education's positive impact on broadening core competencies for conventional healthcare professionals.
- The need for better communication between practitioners of conventional medicine and CAM therapists.
- The potential for improving healthcare coordination.
- The potential impact on increasing the quality and capacity of CAM research.
- The need for improved communication between providers of conventional medical care and patients using CAM.
- The potential for enhancing the quality of care through informed use of CAM.
- The positive impact on enhancing cultural competency.
- The response to governmental, legislative and other mandates.

In Germany, a more conservative approach to CAM has been taken, even though its inclusion in undergraduate training was imposed in 2003 as part of a law on medical education [6–8]. This legal obligation was accompanied by attempts to establish better communication between practitioners of conventional medicine and CAM, but despite some attempts, education in the field is still in its fledgling stages [9].

In 2005, Brinkhaus et al. [10] published the results of a survey in which they had asked directors of German medical schools for their views on CAM. These authors found that 39% of respondents had a positive opinion of CAM, 27% were neutral and 31% viewed it negatively. When asked about integrating CAM into the medical education syllabus, most directors were in favor and only 3% were uncertain. However, integration mainly referred to research (61%) and teaching (59%). A study undertaken in spring 2004 showed that some medical schools had tried to integrate CAM into the teaching schedule [8]. However, even at those universities, which had established CAM education, no common learning program had been developed and agreed [8]. Furthermore, there is still no student textbook covering an agreed syllabus for CAM education in relation to methods and quality issues: that is, evidence-based judgments on therapeutic and diagnostic methods in CAM.

We undertook a questionnaire study which aimed to assess current needs for CAM education by analyzing the views, knowledge and experiences of medical students, office-based general physicians and office-based gynecologists in Germany. We believed that these groups could provide valuable judgments and perspectives on educational requirements and could help to identify the areas of conventional medicine in which CAM could be used most effectively and should therefore be taught more intensively. However, we believe that the development of a curriculum for CAM should be based not only on opinions but also on scientific data.

## 2. Methods

### 2.1. Questionnaires

Based on experiences with earlier studies, we developed two versions of a questionnaire, one for the doctors and the other for medical students. These questionnaires were based on.

- The Attitude toward Alternative Cancer Therapy: Questionnaire which has been shown to be reliable and valid [11–13].
- A modified version of the questionnaires by Newell and Sanson-Fisher [14], and Furnham and McGill [15] which assesses the respondents' knowledge of CAM.

The instruments we developed were alike in terms of questions on views on CAM but differed to some extent in the assessment of demographic data. Copies of the questionnaires can be obtained from one of the authors (K.M.).

### 2.2. Participants and Procedure

All seventh semester medical students attending Giessen University Medical School were given the questionnaire during the summer and winter semesters of 2007. They were asked to complete the questionnaire and were given repeated reminders, if they failed to do so. Questionnaires were also handed out to all office-based general physicians and gynecologist identified from *Yellow Pages* in the cities of Osnabrück, Münster, Bremen, Lingen (Ems), Meppen (Ems), Papenburg and Dorsten, Germany. Practices were visited between April and November 2007 and the intention of the study was explained briefly to the doctor. Those who agreed to fill in the questionnaire were paid a second visit to pick up the completed form. If it had not yet been filled in, the doctor was reminded to do this. We visited the doctors' practices a third time, and if the form had still not been completed we asked them to return it by mail.

### 2.3. Statistical Analysis

SPSS software for Windows, release version 14.01, was used for data management and statistical analyses (bivariate correlation, cross-tabulation). A probability of error <5% was regarded as significant.

### 2.4. Ethical Approval

The project was approved by the ethics committee of the Justus-Liebig-University Giessen on March 15, 2007 (application number 27/07).

## 3. Results

### 3.1. Participants

Between April 2007 and November 2007, we distributed 550 questionnaires—280 to doctors (170 general physicians and 110 gynecologists) and 270 to the medical students. The demographic characteristics of the doctors and students are shown in Table 1. In a few cases, doctors refused to complete the questionnaire or were not available because of sickness or vacation. However, completed questionnaires were returned by 80.4% (217/270) of the students and 78.2% (219/280) of the doctors. This level of response is typical and perfectly adequate for postal questionnaires according to a systematic review of methodology undertaken by Nakash et al. [16] and will not therefore be discussed later.

### 3.2. Sources of Information

**Figure 1:**
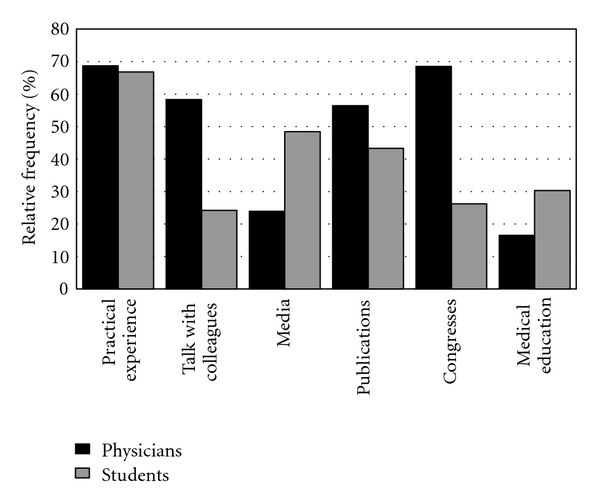
Sources of information regarding CAM.

We asked whether doctors and students had already educated themselves about CAM and which methods of acquiring information they considered the most important. The results showed that 73.8% (160/219) of the doctors and 40% (87/217) of the students had already informed themselves about CAM. The doctors believed that practical experience, congresses, talking with colleagues and publications were the most important sources of information; while students thought that practical experience, information from the media and academic publications were more important.  Figure 1 shows the relative frequencies of those answering “important" and “very important" in relation to the value of the different sources of information.

### 3.3. Levels of Knowledge

**Figure 2:**
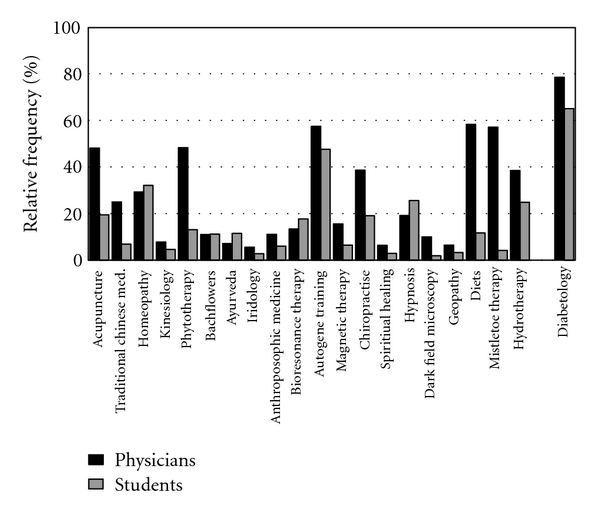
Perceived knowledge of various CAM methods.

Participants were also asked to state subjectively how much they knew about various CAM therapies.  Figure 2 shows the relative frequencies of those answering “good" and “very good" in relation to their knowledge of different CAM therapies. We included diabetology, as a part of conventional medicine, in this section and show results for knowledge of this area for purposes of comparison.  Figure 2 shows that doctors do not feel that they know very much about most CAM therapies. They consider themselves most familiar with diets, autogenic training and mistletoe therapy. Students generally believe that they know much less about all methods, but consider themselves most familiar with autogenic training, homoeopathy and hypnosis. Statistical analysis showed that in most cases the differences between the doctors and students were significant.

### 3.4. Objections to CAM

**Figure 3:**
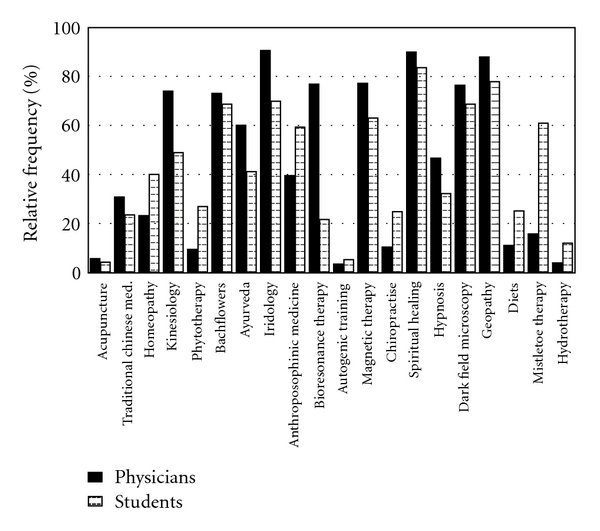
Objections to various CAM methods.

Doctors and students were asked whether they disapproved of certain CAM methods. The results, illustrated in Figure 3, show that the methods most disapproved of by doctors were iridology, dark field microscopy and spiritual healing. Results from the students were similar; they objected most to geopathy, spiritual healing and iridology.

### 3.5. Usefulness of CAM

**Figure 4:**
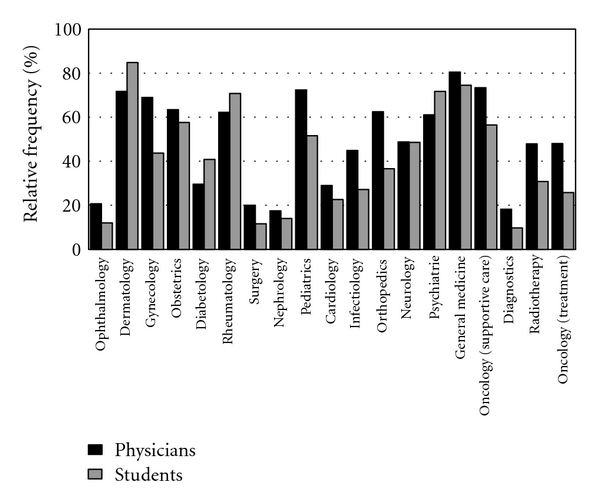
Areas of conventional medicine in which use of CAM are perceived to be reasonable.

**Figure 5:**
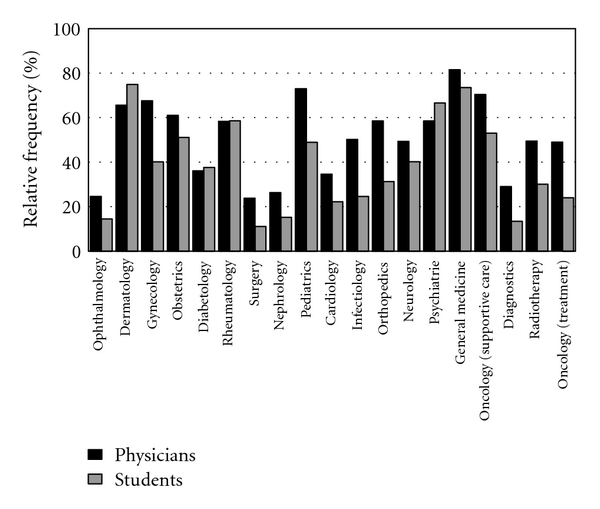
The perceived importance of CAM education in areas of conventional medicine.

We asked participants to indicate those areas of medicine in which they considered CAM to be useful. Doctors believed that CAM was most useful in general medicine, supportive oncology, pediatrics, dermatology and gynecology (Figure 4) and accordingly considered that the teaching of CAM in these areas was important. Students thought that dermatology, general medicine, psychiatry and rheumatology were the areas in which CAM could be used to greatest benefit and therefore recommended that in these areas CAM should be part of the medical curriculum. There was strong correlation between how valuable and reasonable CAM methods were perceived to be in the various areas of conventional medicine and the strength of support for their inclusion in the undergraduate medical curriculum (*r* = 0.496–0.782; *P* < .001) (Figure 5).

### 3.6. Groups of Doctors Compared

We also compared the answers of general physicians and gynecologists and found highly significant correlations between their opinions. Thus, the area of medical practice did not have a major influence on the doctors' opinion on CAM.

### 3.7. Doctors and Students Compared

In Table 2, we summarize doctors' and students' ratings on various aspects of CAM. All differences between doctors and students were statistically significant. In both groups, most respondents believed that including CAM in the undergraduate curriculum at medical school was necessary and useful. In addition, most respondents in both groups were interested in CAM and would have liked additional tuition. Although they did not agree with the statement that “CAM means quackery and charlatanism", both doctors and students strongly agreed that universities should investigate CAM more thoroughly and that CAM should be taught critically. Interestingly, both doctors and students agreed with the statement that doctors are strongly influenced by the interests of pharmaceutical companies. 

## 4. Discussion

The study assessed the experiences and opinions of German doctors and medical students in relation to CAM use in various areas of medicine. We believed that the experience of the doctors with regard to satisfaction or disappointment with conventional medicine or CAM would have influenced their opinions. In general, neither doctors nor students considered themselves to be well informed on CAM and ruled out some CAM methods completely. In some areas of medicine, such as general medicine, use of CAM was considered reasonable, while in others, such as general surgery, they believed that CAM made no sense at all. Thus, doctors and students believed that CAM should be included in the medical education curriculum but that because of the value of CAM varies across medical disciplines and CAM methods, the curriculum should focus on some areas more than others. Unfortunately, it was only possible to analyze the fields of medicine but not various medical situations within each field. This should be done in future studies.

The types of CAM considered to be effective by German physicians differed from those viewed as useful by physicians in the United States and Canada [17, 18]. This finding may result from cultural differences, as has already been suggested [1].

To the best of our knowledge there have been few investigations into medical practitioners' attitudes to CAM in Germany. One interesting difference between Germany and “the rest of the world" is that in the field of oncology. German physicians are the main promoters of CAM despite the fact that many of them admit that they are not well informed about it [1, 19–21]. A very recent publication, which appeared after we completed our study, supports the finding that GPs considered CAM as a reasonable complementary approach within primary care [22].

Studies on the general topic of CAM have been undertaken in many countries worldwide, including the USA, Canada, Great Britain, New Zealand, Turkey, the Netherlands, Israel and Singapore [18, 23–31].

The main findings of these studies are.

- Doctors and students are generally interested in CAM methods [17, 27].
- Doctors are concerned about the effectiveness and possible harmful effects of CAM [27, 28].
- Students admit having little knowledge of CAM [17].
- Value and efficacy of CAM methods differ [30].
- Some doctors had also referred patients for CAM treatments [26].

The fact that CAM is not taught systematically at present may be the consequence of several aspects.

- General reluctance regarding CAM—fear that including CAM education at a university could be interpreted as a sign of acceptance of these methods.
- Lack of knowledge and practical experience.
- Inexperience regarding the conduct and content of lessons—what should be focused on?
- Lack of qualified teachers with a balanced and objective viewpoint who can see beyond the prejudices of CAM protagonists and antagonists.
- The question whether there should be an independent institute for CAM or whether CAM should be taught within the various disciplines.
- The question in how far patients should be treated in this context.

We believe that including CAM methods in the curriculum does not signal the promotion of these methods. Information on the various methods and their potential uses, benefits and risks must be highlighted. After having received the necessary amount of information on CAM and learned how to judge clinical trials, students will be able to make their own judgments on CAM as well as on conventional medicine rather than relying on the judgments of others, including so-called experts. Positive experiences in this field have been reported from Italy [32, 33]. It is worth remembering that with conventional medicine too, not all treatments are actually evidence-based. Students must learn the principles for dealing with CAM methods described by Klimm [34] and Alpert [35]. Basically, these authors recommend the following.

- Maintaining an open mind about all new therapeutic interventions, including CAM.
- Studying CAM in order to be able to advise patients about these methods. Encouraging carefully performed and approximately controlled studies of these new therapies.
- Avoiding of hubristic and arrogant attitudes to alternative medicine as it may prove clinically effective.

Judging CAM methods and conventional medicine by the same scientific and understandable criteria seems to be the right approach, because there has been some evidence that CAM methods may actually extend the possibilities of conventional medicine. For example, a meta-analysis showed that honey is the best treatment for infected wounds and burns [36]. Students will also have to acknowledge that in some areas of medicine, conventional medicine has no simple treatment to offer. A good example is the treatment of labor pain, for which intracutaneous sterile water injections have been found to be effective [37]. This also underscores the necessity of developing CAM research capacity [38].

In conclusion, this study underlines the fact that both German doctors and medical students know about some of the benefits of CAM but would like to be better informed so that they can better manage any CAM questions or problems that arise in their daily practice. In this respect, Germany does not differ from other countries as indicated by studies from other parts of the world [39]. As suggested by Gaster et al. [40] and Pearson et al. [41], a three-step approach to teaching seems reasonable.

1. Teach fundamental CAM competences to give students a framework for learning about CAM.
2. Choose specific content on the basis of evidence, demographics and conditions (what conditions are most appropriate for CAM therapies).
3. Provide students with skills for future learning, including where to find reliable information about CAM and to search scientific literature.

We suggest that alongside the planning of teaching curricula it will be important to develop textbooks on CAM which provide an objective view of the topic. As CAM covers a wide range of diagnostic and therapeutic methods with cover many medical fields, it seems to be important to combine general information on the topic with specific information in the various therapeutic situations. With such a concept physicians will be better prepared for the daily requirements, patients will not be confronted with dangerous and ineffective treatments because of poor knowledge on the topic, and, finally, patients may be able to profit more easily from some benefits of CAM. The results of our study may help to develop educational programs on CAM which are based on the experience of doctors who know about the requirements of day to day practice.

## Figures and Tables

**Table 1:** Characteristics of the two groups of participants.

Characteristic	Doctors (*n* = 219)	Medical students (*n* = 217)
Age, Mean (SD) (years)	48.9 (8.1)	24.8 (2.5)
Gender, *n* (%)		
Male	124 (56.6)	87 (40.1)
Female	95 (43.4)	130 (59.9)
Marital status, *n* (%)		
Single	18 (8.2)	112 (51.6)
Married	188 (85.8)	100 (46.1)
Divorced	13 (5.9)	5 (2.3)
Previous contact with CAM, *n* (%)	219 (100)	86 (39.6)
Years in practice, *n* (%)		
0–10	24 (11)	
11–20	81 (37)	—
21–30	80 (36.5)	
31–50	34 (15.5)	
Academic qualification, *n* (%)		
Medical degree	64 (29.2)	—
Doctorate	147 (67.1)	
Professor	2 (0.9)	
Medical discipline, *n* (%)		
General medicine	132 (60.3)	—
Gynecology	87 (39.3)	
Future personal job perspective, *n* (%)		
Work in hospital	—	117 (53.9)
Work in medical practice		78 (35.9)
Not work as physician		22 (10.2)

**Table 2:** Opinions of students and doctors on statements related to CAM education.